# Does Ultra-Endurance Passion Make Athletes Happy?

**DOI:** 10.3390/sports12060149

**Published:** 2024-05-28

**Authors:** Tatjana Bill, Grégory Dessart, Roberta Antonini Philippe

**Affiliations:** 1 Institut des Sciences du Sport, Faculté des Sciences Sociales et Politiques, Université de Lausanne, 1015 Lausanne, Switzerland; roberta.antoniniphilippe@unil.ch; 2 Institut de Sciences Sociales des Religions, Université de Lausanne, 1015 Lausanne, Switzerland; gregory.dessart@unil.ch

**Keywords:** ultra-endurance, happiness, harmonious passion, obsessive passion, athletic identity, motivation, serious leisure, sport psychology

## Abstract

Sport psychology research of ultra-endurance (UE) athletes focused predominantly on their psychological characteristics, traits, and behaviors. However, their happiness and passion, as well as a unified framework for UE hobby phenomenon, were not sufficiently investigated. This study aims to: (1) identify the main contributors to happiness and passion of non-professional UE athletes; and (2) explore the possible relationships between types of sport passion, motivation, and athletic identity. During data collection, 116 non-professional UE athletes (mean age 43.66 years, SD = 8.97, 16.4% female) responded to an online questionnaire. Statistical analyses revealed that obsessive UE passion (*p* < 0.05) and amotivation (*p* < 0.05) predicted lower levels of happiness. A higher level of obsessive passion was predicted by extrinsic motivation (*p* < 0.005), amotivation (*p* < 0.05), and exclusivity identity (*p* < 0.001); a lower level was predicted by social identity (*p* < 0.05) and years in sports (*p* < 0.05). Weekly training hours and age correlated positively with passion strength, while amotivation was strongly negatively related to training volume. These results indicate that happiness of UE athletes depends on the type of sport passion formed and the quality of the underlying motivation: obsessive passion and amotivation seem to be the main enemies of happiness for UE athletes. This novel finding connecting passion, happiness, and motivation contributes to both a better understanding of the psychology of UE athletes and has practical implications for UE athletes, coaches, athletes’ social circles, and sport psychologists. Due to known maladaptive outcomes of obsessive passion, including its negative impact on overall well-being, health, and now also on happiness, its formation in UE athletes needs to be observed and prevented. While the study shows predictors of obsessive passion and high vs. low obsessive passion, future research should investigate how harmonious passion impacts athletes’ happiness, motivation, and identity. Likewise, research among the UE entourage would help to better understand the social impact of UE as a serious hobby and the formation of UE lifestyles. We also suggest our Temporal Framework for Progressive UE Engagement and Passion, which was further developed based on the results of this study, to be used and validated by sport psychologists.

## 1. Introduction

### 1.1. The Phenomenon of Global UE Sport Popularity

**Figure 1:**
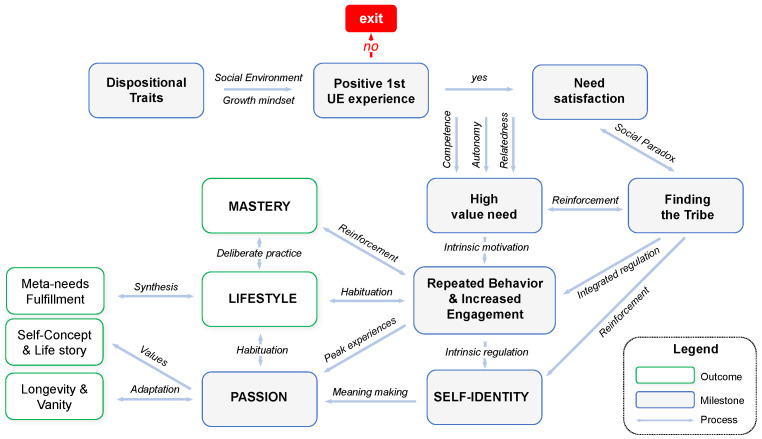
A Temporal Framework for Progressive UE engagement and Passion Development (adapted from [5]).

Ultra-endurance (UE) sports and extreme UE races have been booming globally and the number of participants has been increasing for the past 20 years [1]. In 2023, the International Trail Running Association had a database of 1.7 million unique runners from 22.400 races, which has been growing explosively year on year since its founding in 2013 [2]. A UE race is defined either by a distance exceeding the marathon distance (42.195 km) or racing time exceeding 6 h [3] in sports such as road and trail-running, triathlon, cycling, cross-country skiing, mountaineering, ski touring, open water swimming, etc. With only a small percentage of professional athletes participating, UE sports are the domain of non-professional enthusiasts exercising the “serious leisure hobby” [4]. This growing phenomenon has attracted a lot of academic interest. Researchers studied both the unique physiological markers of UE athletes and their psychological characteristics, traits, and behaviors. Trying to explain the UE phenomenon on a broader social-personal level of lived experiences, in our previous qualitative research of the journey of UE athletes [5] we created a holistic framework of long-term UE engagement and passion development (Figure 1). We aim to enrich this framework in current and future studies with UE athletes.

### 1.2. UE Motivation Research

Starting with the fundamental question of motivation driving non-professional athletes to engage in the demanding hobby of UE training and racing, in addition to job and family obligations, the sport psychology literature is rich. In one pioneering study on the “experience utility” of mountaineering, described as the “worst possible combination of stultifying boredom punctuated by brief moments of terror” [6] (p. 320), the ego involving motives of self-signaling (recognition, prestige, pride, “desire to impress without appearing that one is trying to impress”), as well as intrinsic motives of goal completion, mastery and meaning for life, were hypothesized by the author, himself a mountain climber [6] (p. 322), whereas the “pleasurable consumption utility” was ruled out. Life meaning was identified as the key motivator for Marathon des Sables runners [7,8], Polish ultra-marathoners [9], and German ultra-marathoners [10]. Among Japanese ultra-marathoners, the sense of achievement and goal completion/challenge came out as leading motivators [11]. Achievement motives and self-esteem, life coping and life meaning, followed by health motives were the top ones measured for female American ultra-runners [12]. Among Chinese UE and extreme sports athletes, the motives of mastery, enjoyment, psychological and physical condition, affiliation, others’ expectations, competition/ego, but also vertigo and catharsis were found [13]. According to a systematic review of the literature of psychology of ultra-marathon runners, their key motivating factors are the opportunity to achieve personal goals and feeling a sense of achievement [14]. Despite these results, the literature has not landed on a true consensus or fully grounded theoretical framework to explain the motivation of non-professional UE athletes. Different methodologies were used to study the motivation of athletes: the Motivation of Marathon Runners Scale [7,8,9,10,12], semi-structured interviews [13], Myers-Briggs Type Indicator [11], Sports Mental Toughness Questionnaire [8], and NEO-PI-R (Neuroticism, Extraversion, Openness Personality Inventory revised) questionnaire [8].

### 1.3. Theoretical Foundation and UE Passion Research

This research is grounded in the humanistic paradigm and the Self-Determination Theory of Behavior [15,16], with the latter serving as a meta-theory. According to this theory, humans strive to integrate their behaviors and actions to support and fulfil the three basic psychological needs: competence, relatedness, and autonomy. This integration or internalization of behaviors happens along a continuum with increasing degrees of autonomy and self-determination. Intrinsic motivation happens when the three basic psychological needs are met in one activity, which is performed for its inherent satisfaction [16,17]. The Self-Determination Theory is regarded to be especially fitting to study motivation for physical activity, due to its depth and the different types of regulation involved [18]. The Sport Motivation Scale [19] has been widely applied and is based on the Self-Determination Theory. Many studies using the scale have provided evidence that the more autonomous forms of motivation have important positive implications for athletes’ health and well-being [17]. Specifically, they have been associated with positive outcomes, such as coping strategies [20], positive emotions [19], vitality and well-being [21], and task orientation and engagement in achievement goals [22]. A study on Australian triathletes found a prevalence of both extrinsic and intrinsic motives [23], while American ultra-marathoners were found to have more intrinsic motives (life meaning) than marathon and semi-marathon runners [24].

“Passion is defined as a strong inclination toward an activity that people like, that they find important, and in which they invest time and energy” [25] (p. 757). The Dualistic Model of Passion [25,26,27] defines two types of passion: harmonious and obsessive. These are characterized by the distinctive integration of passion-driven behaviors into one’s identity as well as the specific outcomes they lead to. Harmonious passion (HP) results from an autonomous internalization of passion-related behaviors, with full autonomy and flexibility and tends to fuel motivation, create flow, increase well-being, and provide meaning in life [28]. Obsessive passion (OP) stems from a controlled internalization of behaviors, characterized by open or latent pressures (e.g., parental, social) and external or internal contingences (e.g., scholarship, awards, self-esteem) linked to the activity, leading to less adaptive and sometimes even maladaptive outcomes, such as negative emotions and conflicts with other aspects of life [28,29].

The measurement tool of dualistic passion, the Passion Scale, [25] has been applied to, and has unsurprisingly confirmed, the high prevalence of passion in sports. In general, both HP and OP predicted deliberate practice, which is in itself a predictor of performance in sports [26,30]. Many studies agree that both forms of passion lead to adherence to exercise routines and tolerance of increased exercise, including continuation of it despite physical problems. However, due to its compulsive nature, OP was found to be consistently associated with maladaptive outcomes [31,32,33]. More specifically, while HP was positively related to the exercise dependence dimensions of time and tolerance, OP was positively related to all seven exercise dependence dimensions of time, tolerance, withdrawal, continuance, intention effects, lack of control, and reduction in other activities [34]. Obsessively passionate endurance runners were found to be more susceptible to injury [35]. OP was also found to be associated with a lower quality of relationships with teammates [36] and with negative impact on team cohesion [37]. Obsessively passionate athletes were found to thrive in highly competitive environments, such as training camps, while harmoniously passionate individuals preferred less competitive and demanding ones [38]. A study with Spanish ultra-trail runners (elite and non-professional) found OP to be highly present in runners aiming to achieve the top position in a race, whereby harmonious passion was strongly present with runners just wanting to finish the race [39]. Research with older French runners (M = 60.79) found that HP was a positive predictor of vitality, which was a direct positive predictor of life satisfaction, whereas OP was a direct negative predictor of life satisfaction [40]. In summary, passion seems to represent a key ingredient in the engagement in UE sports, and one which comes with a price for obsessively passionate athletes [41].

### 1.4. UE Identity Research

Identity theories emerged as a standalone self-construct in late 1960s and developed into a massive field of research with many sub-branches [42,43,44]. In sports psychology identity research focused mostly on athletic identity, which is defined as the “degree to which an individual identifies with the athlete role, within the framework of a multidimensional self-concept” [45]. The Athletic Identity Measurement Scale has been widely used to evaluate the strength of self-identification with and dedication to sports [46]. It has been mostly used in professional and elite sports and with collegiate athletes. For example, a study on marathon runners [47] found that high athletic identity in marathoners was connected to better athletic performance and more commitment to running, an expanded social network, and led to more frequent experiences of both the positive and negative effects of marathon training. Athletic identity was found to be strongest with the most successful international level athletes and connected with the motivational characteristics of the athlete’s personality: win orientation, competitiveness, and positive (for men) and negative (for women) competitive orientation [48]. However, research also showed that high athletic identity can be a risk factor for poor adjustments to events hindering further athletic advancement and performance, such as injury [49]. Indeed, according to the meta-study on athletic identity, it correlates with both positive (intrinsic motivation/commitment, mastery goal orientation) and negative (negative emotions, body disorder issues) factors [50]. It was also found that athletic identity tends to decrease with age [45,51]. The most recent systematic review of athletic identity research [52] confirmed that higher-achieving athletes reported a higher degree of athletic identity and that athletic identity correlated with positive and negative factors, thus showing some parallels with the passion concept presented above. Using social identity theory, a study with UE gravel cyclists [53] found that social identity had a significant direct relationship with motivations, constraints, and negotiation strategies and a significant indirect effect on planned participation. There is, however, no research to date exploring athletic identity of non-professional UE athletes and its relationship with passion and happiness constructs—a gap to be explored with this research.

### 1.5. Happiness and Well-Being of UE Athletes

Since Aristotle’s eudemonia and his insights into happiness and moral values, there have been three main streams of happiness theories [54,55]: (1) need satisfaction and goal achievement; (2) process and experience of activities; (3) genetic traits and personality disposition. The positive psychology movement took inspiration from all three streams, becoming “the scientific study of positive experiences and positive individual traits, and the institutions that facilitate their development” [56] (p. 630). It defines happiness as a triad of: (1) pleasure or positive emotion (“the pleasant life”); (2) engagement (“the good life”); and (3) meaning (“the meaningful life”) [57].

The concept of happiness is operationalized and measured by subjective well-being, which is defined as person’s cognitive and affective evaluation of their life as a whole [54]. Subjective well-being is generally studied according to three components of: (1) presence of positive emotions (positive affect), (2) absence of negative ones (negative affect), and (3) a cognitive judgement of life satisfaction and fulfilment (cognitive component). Multi-item measures [58,59,60] are used for the broader coverage of each of the three components and also for investigation of correlations among them. One example of such a scale is the Oxford Happiness Questionnaire [61]. In a study comparing positive moods generated by leisure activities using the Oxford Happiness Inventory [62], only sport/exercise resulted in increased happiness.

In serious leisure sports, most research focused on the impact of people´s leisure experiences, including participation in sport, on their well-being [63,64,65], arriving at a general agreement of a positive relationship, in spite of different theoretical approaches [66]. The topic of happiness and subjective well-being among non-professional UE athletes has, however, been dramatically under-researched. One creative approach [67] applied prospect theory to happiness research—as a reward for “torture” in long-distance triathlon. It was found that satisfaction with race outcomes (i.e., goal achievement), such as finishing the race, positively affected happiness after the race. To the best of our knowledge, no study has yet addressed happiness among UE non-professional athletes.

### 1.6. Hypotheses and Study Objectives

Based on the above literature research and our previous qualitative study with non-professional UE athletes [5], the following hypotheses were formulated to be tested quantitatively: (1) HP will be associated with greater intrinsic sports motivation, greater social identity type of athletic identity, and years in sports; (2) OP will be associated with greater extrinsic motivation and greater exclusivity and negative affectivity types of athletic identity; (3) Happiness will be associated with greater HP, intrinsic motivation, lower OP, and extrinsic motivation.

With these hypotheses, the study aims to: (1) identify the main contributors to happiness and passion of non-professional UE athletes and (2) explore the possible relationships between types of sport passion, motivation, and athletic identity.

## 2. Materials and Methods

### 2.1. Study Design

Participants were recruited via the organizers of Marathon du Mont Blanc in France in July 2021, Facebook groups, and the TrainingPeaks coaching platform. A total of 329 international athletes responded to the online questionnaire in English. General instructions were provided to participants on the landing page of the online survey. Participation was voluntary, participation consents were obtained electronically, and no compensation was provided.

The following eligibility criteria were applied: (1) minimum two UE races lasting over 6 h completed in past three years; (2) amateur/non-professional status; (3) answering all questions of the questionnaire (i.e., standardized scales). As a result, after the data cleansing, the final sample of 116 participants was composed, which built the data set.

### 2.2. Measurement Scales and Methods

The main constructs: sport passion (HP, OP, and passion strength), sport motivation (extrinsic, intrinsic, and amotivation), athletic identity (social, exclusive, negative affectivity), happiness and socio-demographic data (age, gender, years in UE sport, weekly training volume in hours, athletic level, number of finished UE competition in past three years).

Athletes’ motivation for their UE sport was assessed using the Sport Motivation Scale (SMS) [19]. This scale measures the strength of motivation and its source (internal vs. external) to uncover the primary reasons for practicing sport. The scale includes seven sub-scales of four questions each. It measures three types of intrinsic motivation: to know, to experience stimulation, and to accomplish. It also measures three types of extrinsic motivation: external, introjected, identified, plus amotivation. Multiple studies, including a meta-analysis [17,19,68], confirmed its internal consistency and construct validity. For analysis purposes, motivation subscales were grouped together into extrinsic and intrinsic sub-scores.

Passion Scale (PS) [25] was used to measure the passion strength for the sport and the type of passion (HP and OP). Participants first were asked to identify their favorite sport and then answer 17 questions from the Passion Scale regarding how they feel about their passionate activity (sport). The Passion Scale has two six-item subscales, each measuring one of two types of passion: harmonious and obsessive passion. These two subscales are accompanied by a five-item subscale measuring passion criteria (equating to passion strength) to distinguish passionate from non-passionate individuals. The Passion Scale has been widely applied to various fields of research: work, sport, education, music, and the arts, and its validity and internal consistency were confirmed [28].

The Athletic Identity Measurement Scale (AIMS) was used to evaluate the level and type of adoption of sport as part of one´s self-identity [46]. With only seven items, the scale is short, with three items attributed to social identity (e.g., “I consider myself an athlete”) and two each to exclusivity identity (e.g., “Sport is the most important part of my life) and negative affectivity (e.g., “I feel bad about myself when I do poorly in sport”). A systematic review and a meta-analysis of more than 100 published studies that used this scale confirmed its validity as well as its initial hypothesis that more engaged athletes should have higher values for athletic identity [52].

The Oxford Happiness Questionnaire (OHQ) [61], which is rooted in positive psychology, was used to measure subjective psychological well-being or perceived happiness. Participants received the guidance to read the 29 questions carefully since some were phrased positively (e.g., “I feel that life is very rewarding”) and some negatively (e.g., “I don’t think I look attractive”), but not to take too long to answer each question. For the calculation of the happiness score, the score for 12 negatively stated questions was reversed. The total happiness score is calculated as the average of all responses. Participants did not see the result of the assessment.

The Sport Motivation Scale, Passion Scale, and Athletic Identity Measurement Scale all use a 7-point Likert scale (from 1 “strongly disagree” to 7 “strongly agree”), whereas Oxford Happiness Questionnaire uses a 6-point Likert scale.

Sociodemographic data were also collected: age, gender, years in ultra-endurance sports, average weekly training hours in the last 3 years, number of competitions lasting over 6 h, athletic level (age-group “finisher” and competitive age-group).

### 2.3. Data Analysis

First, multiple regression analyses were used in order to determine the main contributors to happiness, HP and OP. Potential contributor variables were chosen based on the main concepts of interest for this study: passion types, motivation, identity, happiness. Second, Spearman’s rho correlations and Mann–Whitney U tests were used to explore the existing relationships between sociodemographic variables and motivation and passion types, respectively. Third, OP was also examined using a median split, and Mann–Whitney U tests were conducted to look into differences between low- and high-OP in regard to their relationships with sociodemographic data, sports identity, and sports motivation.

The main assumptions for carrying out multiple regression analyses, such as no multi-collinearity and multivariate normality of residuals, were met. For further analyses, Shapiro–Wilk tests revealed that the values of most variables were not normally distributed, therefore non-parametric statistical analyses were conducted for these analyses (i.e., Spearman’s rho correlations and Mann–Whitney U tests).

All tests were performed using SPSS (version 28).

## 3. Results

### 3.1. Descriptive Statistics

Descriptive statistics for 116 UE athletes are shown in Table 1. They reveal the heterogeneity of the data set, which is inherent to the sample of non-professional UE athletes with very diverse ages, training methods, and competition participation frequency. At the same time, the data set is rather representative of the UE population in terms of gender, age, and education level.

In addition to the data presented in Table 1, our dataset consisted of 19 (16.4%) female and 97 (83.6%) male respondents. They reported their athletic level as finishers (68.1%) or competitive (non-professional) athletes (31.9%). Professional athletes were excluded from the study due to the abovementioned eligibility criteria.

Regarding family composition: 19.8% live alone, 79.3% with another adult individual, and 0.9% live with four other adults. Regarding children living in the same household: 39.3% reported no children living in the same household, 15% one child, 25.2% two children, 15.9% three children, 3.7% four children, and 0.9% five children.

Main UE sports (non-exclusive): running 91.5%, cycling 6.9%, triathlon 2.6%, skiing 1.8%, canoeing 0.9%. Among runners, 68.42% indicated the main sport being trail running; for the remaining 23.1%, it was not clearly stated. Additional non-UE sport activities included football 1.8% and karate 0.9%.

Regarding education, the following were reported: 53.4% Master’s degree, 15.5% doctorate degree, 11.2% Bachelor’s degree, 7.8% professional degree, 5.2% high school degree, 4.3% trade/technical/vocational training, 2.6% college degree.

### 3.2. Main Contributors to Happiness, Harmonious Passion and Obsessive Passion

To test this study´s hypotheses, three multiple linear regression analyses were carried out. The first model (Table 2), with happiness as the dependent variable, explained 36.4% (adjusted R-square) of the variance of happiness, *F* (15, 100) = 3.816, *p* < 0.001. Significant individual contributions of the predictors showed that OP (*p* = 0.035) and Amotivation (*p* = 0.013) predicted lower levels of happiness. No significant contribution of either intrinsic or extrinsic types of motivation types could be revealed.

The second model (Table 3), with HP as the dependent variable, explained 23.7% of the variance (adjusted R-square) of HP, *F*(12, 103) = 2.661, *p* = 0.004. Significant individual contributions of the predictors showed that SMS Amotivation (*p* = 0.013) predicted lower levels of HP and that—reaching near-significance—SMS Intrinsic motivation combined score (*p* = 0.052) predicted higher levels of HP.

The third model (Table 4) with OP as the dependent variable explained 52.9% of the variance (adjusted R-square) of OP, *F* (12, 103) = 9.649, *p* < 0.001. Significant individual contributions of the predictors showed that SMS Extrinsic motivation combined score (*p* = 0.003), SMS amotivation (*p* = 0.049), and AIMS Exclusivity athletic identity (*p* < 0.001) predicted higher levels of OP, and that AIMS Social athletic identity (*p* = 0.011) as well as years in sports (*p* = 0.013) predicted lower levels of OP.

### 3.3. Motivation, Passion and Sociodemographic Data

Non-parametric analyses (Spearman’s rho correlations and Mann–Whitney U tests) were conducted to identify significant relationships between sports passion types (HP and OP) and passion strength, on the one hand, and sports motivation (extrinsic, intrinsic, amotivation) and sociodemographic variables (age, gender, years in sports, UE competitions completed, average weekly training hours in the last 3 years) on the other hand.

Regarding sports passion, a significant positive relationship was found between passion strength and age (r = −0.199, *p* = 0.032) as well as with average weekly training volume in the last 3 years (r = 0.36, *p* < 0.001). No significant relationship could be observed between socio-demographic variables and HP or OP, respectively. Additionally, Mann–Whitney U tests were carried out in order to test for gender differences, but no significant differences could be observed between female and male participants with regard to passion strength or passion types (HP, OP).

As for motivation types, a significant negative relationship was found between SMS amotivation and the average weekly training hours in the last 3 years (r = −0.22, *p* = 0.015). Additionally, Mann–Whitney U tests were conducted to test for gender differences, but no significant differences could be observed between female and male participants with regard to motivation types.

### 3.4. Passion Types of UE Athletes

Given the mixed nature of passion and a partial overlap between harmonious and obsessive passion scores, the decision was made to focus on obsessive passion for analysis purposes. The median (Mdn) for OP was 19.5, and the sample was split into two categories: low OP (cumulative percent: up to 50.0; range: 6–19) and high OP (cumulative percent: 52.6 through highest; range: 20–39). Mann–Whitney U tests were conducted to identify whether a sample split based on OP lower and higher scores could lead to significant differences on several variables of interest. The following seven variables showed statistically significant differences:

1. The number of children in one’s household for the high-OP group (Mdn = 1) was lower than for the low-OP group (Mdn = 2). A Mann–Whitney U test indicated that the difference was statistically significant, U (N_high-OP_ = 53, N_low-OP_ = 54) = 1017.50, z = −2.69, *p* = 0.007.

2. The AIMS Social identity scores of the high-OP group (Mdn = 14.0) were higher than those of the low-OP group (Mdn = 13.0). A Mann–Whitney U test indicated that the difference was statistically significant, U(N_high-OP_ = 58, N_low-OP_ = 58) = 1249.00, z = −2.40, *p* = 0.016.

3. The AIMS Exclusivity identity scores of the high-OP group (Mdn = 9.0) were higher than those of the low-OP group (Mdn = 6.0). A Mann–Whitney U test indicated that the difference was statistically significant, U(N_high-OP_ = 58, N_low-OP_ = 58) = 777.00, z = −5.02, *p* < 0.001.

4. The AIMS Negative Affectivity scores of the high-OP group (Mdn = 11.0) were higher than those of the low-OP group (Mdn = 8.0). A Mann–Whitney U test indicated that the difference was statistically significant, U(N_high-OP_ = 58, N_low-OP_ = 58) = 871.50, z = −4.51, *p* < 0.001.

5. The SMS Intrinsic motivation scores of the high-OP group (Mdn = 67.0) were higher than those of the low-OP group (Mdn = 60.0). A Mann–Whitney U test indicated that the difference was statistically significant, U(N_high-OP_ = 58, N_low-OP_ = 58) = 1145.50, z = −2.96, *p* = 0.003.

6. The SMS Extrinsic motivation scores of the high-OP group (Mdn = 58.5) were higher than those of the low-OP group (Mdn = 49.5). A Mann–Whitney U test indicated that the difference was statistically significant, U(N_high-OP_ = 58, N_low-OP_ = 58) = 802.50, z = −4.86, *p* < 0.001.

## 4. Discussion

Academic research in the sport psychology of UE disciplines has traditionally set a very specific focus on the psychological traits and characteristics of athletes from professional or collegiate samples. It has largely overlooked non-professional athletes as well as the concept of happiness. The current study aimed to shed light on UE sports being practiced as a serious leisure activity, hence covering the main type of athletes that are found in the general population—the non-professional ones.

This study investigated the happiness and passion of non-professional UE athletes and explored the relationships between two types of passion (harmonious and obsessive), three types of sports motivation (intrinsic, extrinsic and amotivation), three types of athletic identity (social, exclusivity, negative affectivity), and available demographic data. In addition, the study contributed to a better and more holistic understanding of the psychology of non-professional UE athletes and the phenomenon of UE in society and served to verify and enrich the temporal framework of UE engagement and passion development, as presented in Figure 1.

Hypotheses were partially or not confirmed, while some novel and unpredicted insights were generated. Hypothesis 1: “HP will be associated with greater intrinsic sports motivation, greater social identity type of athletic identity, and years in sports” was not confirmed. Although no significant relationship was shown between HP and social identity, a negative and significant relationship was observed between social identity and OP and years in sports and OP. Hypothesis 2: “OP will be associated with greater extrinsic motivation and greater exclusivity and negative affectivity types of athletic identity” was mostly confirmed: OP was positively associated with greater extrinsic motivation and exclusivity type of athletic identity. Hypothesis 3: “Happiness will be associated with greater HP, intrinsic motivation, lower OP, and extrinsic motivation” was partially confirmed: happiness was associated with lower OP. This finding is the most novel and notable in terms of the contribution of this study to the sport psychology of UE athletes. No significant relationship could be found between happiness and HP, nor between HP and years in UE sports. However, years in UE sports was significantly and negatively associated with OP. Unpredicted was the significant role played by amotivation, which showed a positive relationship with OP and a negative one with both HP and happiness.

The rather negative role of OP is supported by many studies demonstrating the maladaptive outcomes of OP and its negative contribution to well-being due to the conflicts and contingencies inherent to it [29,37,69,70]. In sports, being obsessively passionate can lead to injuries, burn-out, and increased risky and unethical behaviours [27]. This list of maladaptive outcomes can now be expanded; a lower level of happiness can be added for non-professional athletes in UE sports and potentially for other groups of athletes. Interestingly, the present study also found that amotivation predicted lower levels of happiness. Without motivation there cannot be any passion, and passion is confirmed to be an important ingredient to human well-being [71]. While studies on the general population demonstrated a positive correlation between HP and happiness/well-being [71,72], and also a positive relationship between subjective happiness and engaging in physically active leisure [65], no such relationship could be observed in the current study, which may be due to the extremely ambivalent nature of UE sports experience. What can be stated clearly, however, is that having no (or no more) passion for UE sports or being obsessively passionate about it has a negative impact on the happiness of athletes.

The present study also highlighted relationships between OP, motivation, and athletic identity. First, OP showed a positive relationship with extrinsic motivation. This is in line with past research, showing that while all sources of motivation can lead to the emergence of passion, the development of OP mostly follows extrinsic motivations and non-autonomous internalization [25,73]. The present study also found that OP was associated with greater exclusivity in athletic identity. This type of athletic identity is characterized by the following statements: “Sport is the most important part of my life” and “I spend more time thinking about sport than anything else”. At the same time, social identity (“I consider myself an athlete”, “I have many goals related to sport”, and “Most of my friends are athletes”) predicted lower levels of OP. Based on these findings, it can be posited that when exclusivity athletic identity is coupled with extrinsic motivation (“I practice sport because people around me think that it’s important to be in shape”), the development of OP seems to be likely, which in turn predicts lower happiness, as presented above. With these three constructs a psychological model of OP in UE sports for non-professional athletes can be suggested: being extrinsically motivated with exclusive priority on UE sports in life is associated with OP, which predicts lower happiness of UE athletes.

Exploring OP further by contrasting two groups of participants—each one displaying higher or lower levels of OP, respectively–the following observations could be made about higher levels of OP: (1) higher scores across all types of identity (social, exclusivity, and negative affectivity, (2) higher scores on both intrinsic and extrinsic motivations compared to athletes with lower OP scores, (3) less children in the household. To sum up: high OP seems to create quite an extreme UE athlete profile. It can be a profile of athletes who are very competitive, aspire to perform on professional level, and have a lot of ego invested in their UE hobby. It has been established that both HP and OP contribute to performance via their link to deliberate practice [26], which takes time and dedication [74]. The current study also found a positive correlation of passion strength with the age of the athletes and their training hours. At the same time, amotivation correlated negatively with training hours. Amotivation also predicted higher levels of OP and lower levels of happiness. Amotivation as a maladaptive or poor form of motivation regulation, and was shown to be correlated with OP in a meta-study on the dualistic model of passion [32]. The exact mechanisms of amotivation formation and impact in UE sport are likely to relate to the broader topics of psychological well-being and mental health in sports. The latter has been a hugely debated topic in the media and academia in recent years, especially in relation to professional athletes. In spite of the general agreement among scholars that subjective well-being and vitality are increased by many types of exercise [75], there is also evidence of higher depression rates among extreme athletes. In addition, one study found that 20% of ultramarathoners screened positive for exercise addiction concerns compared to 0.5% for the general population and 3% for habitual exercisers [76].

**Figure 2:**
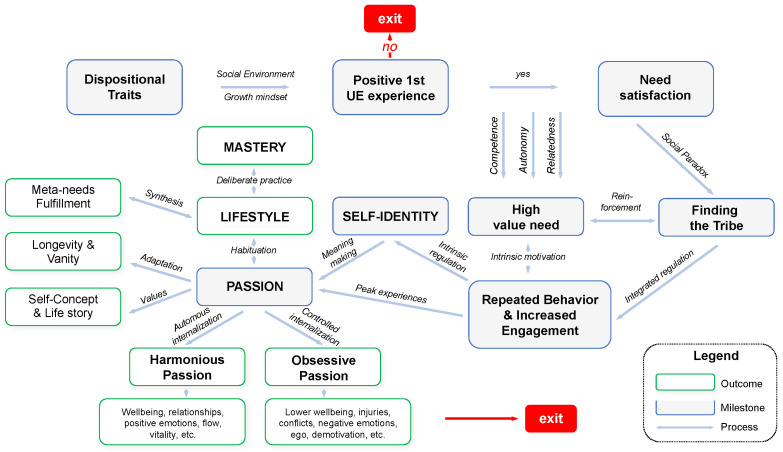
Expanded Temporal Framework for Progressive UE Engagement and Passion Development.

The Temporal Framework for Progressive UE Engagement and Passion Development needs further validation too. However, already at this stage it can be conducive for taking on a broader and more holistic view on UE athletes, considering the long-term nature of their engagement and its major impact on self-identity and lifestyle. Based on the current study, more granularity of the passion construct was added to the framework (Figure 2). Key areas of outcomes for both HP and OP and an indication of the possibility of terminating the UE engagement in case of OP were also added. The academic community and sport psychologists are invited to discuss this framework, which can be used as a map for research on UE engagement.

This study has several practical implications for UE athletes, their coaches, social circles, and sport psychologists. Knowing the maladaptive outcomes of OP, including its negative impact on overall well-being and happiness, conditions need to be created to foster the formation of HP, thus enabling athletes to excel now and to thrive in the long term. Encouraging autonomy, supporting competence development, avoiding that autonomy-limiting external contingencies are attached to the hobby and its results, and promoting broader concepts of identity are key for the optimal functioning of UE athletes.

From an applied perspective, the findings provide some insight into how athletes themselves may enhance their happiness. To this end, athletes should seek to engage in their sport in an open-minded and non-defensive manner (as characterized by harmonious passion) to foster balance and perhaps better performance. UE sports might benefit from being assisted by multidisciplinary teams of professionals specializing in types of physical and psychological support that could help promote harmonious passionate engagement in the activity.

## 5. Limitations and Future Research

This study has several limitations. First, the sample of 116 athletes was predominantly composed of ultra-trail runners, thereby representing a very heterogeneous group of athletes. Looking into wider UE populations (e.g., cyclists, triathletes, swimmers, cross-country skiers) while being more specific with sampling might lead to more precise and target group specific observations. Second, the design of the study was correlational, thus leading to caution about any causal assumptions. Third, the cross-sectional nature of this study did not allow us to consider the usually long timeline of UE passion development. Similar to a UE race, the journey of UE passion is an extremely long one and should therefore also be studied according to longitudinal research designs. Lastly, female individuals were underrepresented in the current study, and even though no gender differences could be recorded it might be helpful to gather samples where female UE athletes are better represented.

Future research should investigate how HP impacts athletes´ happiness, motivation, and their identity. Also, more research on the impact of OP in the short- and long-term should be conducted. The close social circle of UE athletes (spouses and partners) should also be considered to better understand the social impact of UE as a serious hobby and the formation of UE lifestyles. Another important line of research should take athletes´ mental health into account in relation to passion types, motivation, and athletic identity. This will enable field-based sport psychologists to detect and prevent potentially negative courses of development in UE athletes.

## 6. Conclusions

Does UE passion make UE athletes happy? Our answer is: “It depends”. It depends on the type of passion formed and the quality of the underlying motivation. Hypotheses were partially supported: harmonious passion did not appear to play a major role, although obsessive passion and amotivation seem to represent the main psychological hurdles to happiness of UE athletes. Extrinsic motivation, amotivation, exclusivity type of athletic identity predicted higher obsessive passion. In other words, being extrinsically motivated or demotivated and placing exclusive priority on UE sport in life tends to be associated with lower happiness. Lessons learned for UE athletes and possibly beyond: while all types of passion might lead to performance, being obsessively passionate can be detrimental to one´s happiness.

## Figures and Tables

**Table 1:** Descriptive Statistics of Participants.

Baseline Characteristics	N	Min	Max	Mean	SD	Variance
Age	116	24	64	43.66	8.97	80.47
Years in sports	116	2	51	15.4	11.5	131.4
Avg weekly training volume last 3 years	116	1	16	6.85	3.38	11.4
Nr of competitions over 6 h completed	116	2	25	6.53	4.5	20.22
PS Obsessive Passion (OP)	116	6	39	20.36	7.76	60.21
PS Harmonious Passion (HP)	116	24	42	35.45	3.68	13.54
PS Passion Criteria	116	17	35	29.78	4.18	17.46
AIMS Social	116	3.0	21.0	13.37	3.43	11.75
AIMS Exclusivity	116	2.0	14.0	7.71	3.15	9.93
AIMS Negative affectivity	116	2.0	14.0	9.41	2.81	7.88
AIMS Total score	116	12.0	46.0	30.50	7.33	53.75
OHQ Total score	116	75.0	163.0	130.55	18.59	345.57
SMS Intrinsic combined score	116	36.0	83.0	63.0	9.64	93.03
SMS Extrinsic combined score	116	16.0	73.0	53.55	10.47	109.64
SMS Amotivation	116	4.0	23.0	10.67	4.46	19.87

Abbreviations: PS—passion strength, OP—obsessive passion, HP—harmonious passion, AIMS—athletic identity measurement scale, OHQ—Oxford Happiness Questionnaire, SMS—sport motivation scale.

**Table 2:** Multiple Regression Predicting Happiness.

Predictors	*b*	*t*	*β*
(Constant)	90.851 **	4.449	
SMS Intrinsic motivation	0.295	1.385	0.153
SMS Extrinsic motivation	0.134	0.597	0.075
SMS Amotivation	−1.031 *	−2.534	−0.247
AIMS Social identity	0.069	0.107	0.013
AIMS Exclusivity identity	−1.024	−1.457	−0.174
AIMS Negative affectivity identity	−1.264	−1.807	−0.191
Age	−0.127	−0.592	−0.061
Gender	3.417	0.797	0.068
PS Obsessive Passion	−0.596 *	−2.132	−0.249
PS Harmonious Passion	0.890	1.799	0.176
PS Passion Criteria	0.899	1.638	0.202
Years in sports	−0.080	−0.528	−0.049
Avg weekly training volume last 3 years	0.212	0.405	0.405
Number of competitions over 6 h completed	0.042	0.383	0.035
Athletic level	−1.464	−0.359	−0.359

* *p* < 0.05; ** *p* < 0.001.

**Table 3:** Multiple Regression Predicting Harmonious Passion.

Predictors	*b*	*t*	*β*
(Constant)	26.452 **	7.960	
SMS Intrinsic motivation	0.085	1.962	0.222
SMS Extrinsic motivation	0.050	1.094	0.141
SMS Amotivation	−0.210 *	−2.536	−0.254
AIMS Social identity	0.022	0.171	0.021
AIMS Exclusivity identity	0.190	1.463	0.162
AIMS Negative affectivity	−0.075	−0.506	−0.057
Age	0.045	1.000	0.110
Gender	−0.456	−0.501	−0.046
Years in sports	−0.004	−0.118	−0.011
Avg weekly training volume last 3 years	0.053	0.477	0.049
Number of competitions over 6 h completed	0.033	1.423	0.137
Athletic level	−0.463	−0.538	−0.059

* *p* < 0.05; ** *p* < 0.001.

**Table 4:** Multiple Regression Predicting Obsessive Passion.

Predictors	*b*	*t*	*β*
(Constant)	−1.634	−0.297	
SMS Intrinsic motivation	0.080	1.117	0.099
SMS Extrinsic motivation	0.228 **	3.035	0.308
SMS Amotivation	0.273 *	1.995	0.157
AIMS Social identity	−0.559 *	−2.602	−0.247
AIMS Exclusivity identity	1.047 ***	4.876	0.425
AIMS Negative affectivity	0.220	0.897	0.079
Age	−0.017	−0.230	−0.020
Gender	0.220	0.146	0.011
Years in sports	−0.131 *	−2.514	−0.193
Avg weekly training volume last 3 years	0.120	0.654	0.052
Number of competitions over 6 h completed	0.028	0.725	0.055
Athletic level	1.628	1.142	0.098

* *p* < 0.02; ** *p* < 0.005; *** *p* < 0.001.

## Data Availability

The data that support the findings of this study are available from the corresponding author upon reasonable request. The anonymized research data is stored on an academic server based in Switzerland in accordance with the Swiss Federal act on data protection (FADP 235.1) and the Canton of Vaud Act on data protection (LPrD 172.65).
